# Performance of the Quidel Sofia rapid influenza diagnostic test during the 2012–2013 and 2013–2014 influenza seasons

**DOI:** 10.1111/irv.12380

**Published:** 2016-03-23

**Authors:** Peter E. Kammerer, Jennifer M. Radin, Anthony W. Hawksworth, Chris A. Myers, Gary T. Brice

**Affiliations:** ^1^ Operational Infectious Diseases DepartmentNaval Health Research CenterSan DiegoCAUSA

**Keywords:** Influenza, rapid influenza diagnostic test, US–Mexico border

## Abstract

The Quidel Sofia Influenza A+B Fluorescent Immunoassay was used to test nasal swab specimens from patients with influenza‐like illness at US–Mexico border‐area clinics in the 2012–2013 and 2013–2014 influenza seasons. Compared with real‐time reverse transcription polymerase chain reaction, the overall sensitivities and specificities were 83% and 81%, and 62% and 93%, respectively.

## Introduction

Influenza infections are an important cause of morbidity and mortality worldwide in pediatric and adult patients.^1^, ^2^, ^3^ Early identification of influenza virus as the cause of a respiratory illness is essential for managing patients, facilitating appropriate use of antiviral medications, avoiding unnecessary antibiotic prescriptions, and implementing infection control measures.^1^, ^2^, ^4^, ^5^

Rapid influenza diagnostic tests (RIDTs) are point‐of‐care tests that detect influenza viral antigens with quick results, usually 30 minutes or less. The Sofia Influenza A+B Fluorescent Immunoassay (Quidel Corporation, San Diego, CA) uses immunofluorescence‐based lateral‐flow technology to identify viral nucleoprotein antigens in nasal swabs, nasopharyngeal (NP) swabs, and NP aspirates/washes. The Sofia instrument scans the test strip and displays the result, eliminating subjective test interpretation by an individual.^4^, ^6^, ^7^ The Sofia instrument has a modem available that uploads the number and type of tests done and their results to a server every 24 hours, allowing real‐time tracking of testing performed.

This report compares the performance of the Sofia RIDT and the Centers for Disease Control and Prevention (CDC) Human Influenza Virus Real‐Time RT‐PCR Diagnostic Panel (Influenza A/B Typing Kit). The Sofia RIDT, cleared by the US Food and Drug Administration (FDA), received a Clinical Laboratory Improvement Amendments Certificate of Waiver in December 2014. The FDA‐cleared real‐time reverse transcription polymerase chain reaction (RT‐PCR) panel testing was conducted in our College of American Pathologists‐certified laboratory at Naval Health Research Center (NHRC).

## Methods

Since 2004, the NHRC Operational Infectious Diseases Department has collaborated with the CDC Binational Border Infectious Disease Surveillance program, the County of San Diego Health and Human Services Agency, Imperial County Public Health Department, and California Department of Public Health to provide laboratory‐based influenza‐like illness (ILI) surveillance in California clinics near the US–Mexico border. This research has been classified as public health surveillance (non‐human subjects research) by the NHRC Institutional Review Board and as such does not require written informed consent.

In 2012–2013, NHRC provided Sofia instruments to three of four border‐area outpatient clinics. The remaining clinic used the Quidel QuickVue A+B RIDT, which allowed a comparison of the two RIDTs in the same influenza season. In 2013–2014, all four clinics used the Sofia instrument. The ILI case definition of temperature >100°F or subjective fever (patient‐reported fever) with either a cough or a sore throat in the absence of a known cause other than influenza was used at all four clinics. Patients with ILI had two nasal swabs taken, one with the RIDT‐supplied swab, and one with a flocked swab that was subsequently placed into universal transport medium (UTM). Additionally, a standard case report form was used at all four sites to collect data on the patient population at each site.

The UTM swab was used for RT‐PCR testing, the standard used for comparison with the RIDT results. Sofia RIDT testing was done immediately according to the manufacturer's instructions. The nasal swab in UTM was frozen at −70°C and transported to NHRC. These specimens were aliquoted and tested with the RT‐PCR Influenza A/B Typing Kit.

Age group and sex distributions were compared across populations using chi‐squared tests. Sensitivity, specificity, and positive and negative predictive values were calculated with exact (Clopper–Pearson) 95% confidence intervals. All analyses were conducted using SAS software, version 9·3 (SAS Institute Inc., Cary, NC).

## Results

While the majority (>70%) of patients at all clinics were under the age of 25 years, there were some significant age distribution differences across the three populations. The QuickVue RIDT population had a higher proportion of patients aged 0–4 and 5–24 years than the 2012–2013 Sofia RIDT population (*P* < 0·0001). The 2013–2014 Sofia population had a lower proportion of participants in the 0–4 and 5–24 age groups compared with the 2012–2013 Sofia (*P* < 0·0001) and QuickVue (*P* < 0·0001) populations. There were no significant differences between the other age groups in either influenza season. Sex distribution was not significantly different across the 3 populations (Table 1).

**Table 1:** Comparison of characteristics of sofia and quickvue populations

Characteristic	QuickVue A+B (2012–13)	Sofia (2012–13)	Sofia (2013–14)	*P* value
	N (%)	N (%)		
Male	40 (39)	179 (49)	213 (47)	0·24
Age group, years				<0·0001
0–4	28 (28)	147 (40)	176 (35)	
5–24	43 (43)	194 (53)	225 (45)	
25–49	19 (19)	16 (4)	60 (12)	
50–64	7 (7)	8 (2)	28 (6)	
65+	4 (4)	5 (1)	10 (2)	

In 2012–2013, the three clinics using the Sofia RIDT tested 372 ILI patients, of whom 36·9% tested positive for influenza virus by RT‐ PCR (20·3% influenza A, 16·6% influenza B). The clinic using the QuickVue A+B RIDT tested 102 ILI patients, of whom 51.9% tested positive for influenza virus by RT‐PCR (28·3% influenza A, 23·6% influenza B). Overall sensitivities and specificities for the Sofia RIDT were 83% and 81%, respectively, and 59% and 100%, respectively, for the QuickVue A+B RIDT. Influenza A sensitivities and specificities for the Sofia RIDT and the QuickVue A+B RIDT were 82% and 98%, and 57% and 100%, respectively. Influenza B sensitivities and specificities for the Sofia RIDT and the QuickVue A+B RIDT were 84% and 83%, and 62% and 100%, respectively (Table 2).

**Table 2:** Sensitivity, specificity, and positive and negative predictive values estimates by rapid influenza diagnostic test type^a^^,^ b

Rapid test	Category	Sensitivity	Specificity	Positive predictive value	Negative predictive value
Sofia (2012–13)	Overall	83 (76–88)	81 (75–86)	80 (73–85)	84 (78–89)
	Influenza A	82 (73–89)	98 (94–99)	95 (88–99)	90 (84–94)
	Influenza B	84 (74–91)	83 (77–88)	67 (57–76)	92 (87–96)
QuickVue (2012–13)	Overall	59 (46–71)	100 (91–100)	100 (90–100)	62 (49–73)
	Influenza A	57 (39–74)	100 (91–100)	100 (83–100)	73 (59–84)
	Influenza B	62 (40–80)	100 (91–100)	100 (79–100)	80 (66–90)
Sofia (2013–14)	Overall	62 (55–70)	93 (89–95)	83 (75–89)	82 (78–86)
	Influenza A	71 (60–80)	97 (95–99)	89 (80–95)	92 (88–94)
	Influenza B	53 (42–64)	95 (92–97)	75 (62–85)	88 (84–92)

a Values in parentheses are 95% confidence intervals.

b Real‐time reverse transcription polymerase chain reaction testing was used as the gold standard.

In 2013–2014, all four clinics used the Sofia RIDT and tested 499 patients, of whom 30·1% tested influenza RT‐PCR positive (15·0% influenza A, 15·1% influenza B). Overall sensitivity and specificity for the Sofia RIDT were 62% and 93%, respectively. Sensitivities and specificities for the Sofia RIDT for influenza A and influenza B were 71% and 97%, and 53% and 95%, respectively (Table 2).

The PCR cycle threshold (CT) values of false‐negative and true‐positive RIDT results were compared. The CT values were significantly higher (i.e., lower viral RNA titer) with the 2012–2013 QuickVue, 2012–2013 Sofia, and 2013–2014 Sofia influenza B false‐negative results than the same rapid tests' true‐positive CT values (*P* < 0·05). The RT‐PCR CT values for the Sofia 2013–2014 influenza A results were not significantly different between the false‐negative and true‐positive rapid results (results not shown).

## Discussion

In 2012–2013, the Quidel Sofia had higher sensitivity than the Quidel QuickVue test for influenza A+B, influenza A, and influenza B. However, the Quidel Sofia had a decreased specificity for influenza A+B and influenza B compared with the QuickVue RIDT. False‐positive influenza B results, which were observed with the Quidel Sofia, resulted in decreased specificity. These results are similar to those found in some studies 1, 5, 6, 7, 8 and an improvement on the sensitivity found in other studies.^2^, ^3^, ^4^, ^9^, ^10^, ^11^, ^12^, ^13^ The higher sensitivity of the Sofia RIDT made it more likely for clinicians to detect influenza in 2012–2013, compared with the Quidel QuickVue RIDT, while maintaining a high specificity, especially for influenza A.

In 2013–2014, Quidel Sofia results were very similar to what we observed for the Quidel QuickVue in the same population over the last seven influenza seasons: poor sensitivity and very good specificity P.E. Kammerer (unpublished data). The Quidel Sofia's specificity improved to levels historically seen with the Quidel QuickVue A+B in our ILI surveillance. However, the overall sensitivity decreased to similar levels as seen with the Quidel QuickVue A+B in past influenza seasons P.E. Kammerer (unpublished data). This decrease was mostly due to a decrease in the influenza B sensitivity. As a result of the higher specificity, a positive Quidel Sofia result in 2013–2014 was less likely to be false positive. However, due to low sensitivity, a negative result combined with a clinical picture of influenza should not have precluded treatment for influenza.

Some factors that may decrease the sensitivity of the Sofia RIDT are improper swabbing technique,^9^ taking samples later in the disease course when viral titers are often lower,^2^, ^5^, ^9^, ^13^ and sampling from an older population (children have higher viral shedding and viral titers, which may have contributed to the decreased sensitivity seen in 2013–2014).^2^, ^3^, ^8^, ^10^, ^11^, ^13^ These factors are thought to be the most likely to have affected our results. Other factors may include time elapsed before testing,^2^ variation in sampling technique between sites, variation with influenza subtype,^12^, ^13^ choice of reference test (RT‐PCR vs viral culture),^7^, ^9^ specimen type,^10^, ^11^ and storage and transport conditions.^3^, ^9^, ^13^

The differences in the Sofia's sensitivity and specificity between the two influenza seasons (2012–2013 and 2013–2014) merit continued monitoring of the performance of the Sofia RIDT during future influenza seasons.

## Disclaimer

The views expressed in this presentation are those of the authors and do not necessarily reflect the official policy or position of the Department of the Navy, Department of Defense, or the U.S. Government. Approved for public release; distribution is unlimited. This research has been conducted in compliance with all applicable federal regulations governing the protection of human subjects in research. U.S. Government Work (17 U.S.C. § 105). Not copyrighted in the U.S.

## Financial support

Funding was provided in part by the Armed Forces Health Surveillance Center, the Field Forward Diagnostics program of the Defense Threat Reduction Agency, and the Epidemiology and Laboratory Capacity for Infectious Diseases Cooperative Agreement (1U50CK000410) from the US Centers for Disease Control and Prevention.

## References

[1] Dunn J , Obuekwe J , Baun T , Rogers J , Patel T , Snow L . Prompt detection of influenza A and B viruses using the BD Veritor^™^ System Flu A + B, Quidel^®^ Influenza A +B FIA, and Alere BinaxNOW^®^ Influenza A&B compared to real‐time reverse transcriptase‐polymerase chain reaction (RT‐PCR). Diagn Microbiol Infect Dis 2014; 79:10–13. 24582581 10.1016/j.diagmicrobio.2014.01.018

[2] Chartrand C , Leeflang M , Minion J , Brewer T Pai M . Accuracy of rapid influenza diagnostic tests: a meta‐analysis. Ann Intern Med 2012; 156:500–511. 22371850 10.7326/0003-4819-156-7-201204030-00403

[3] Gao F , Loring C , Laviolette M *et al* Detection of 2009 pandemic influenza A(H1N1) virus infection in different age groups by using rapid influenza diagnostic tests. Influenza Other Respir Viruses 2012; 6:e30–e34. 22114876 10.1111/j.1750-2659.2011.00313.x PMC4941676

[4] Chu H , Lofgren E , Halloran M *et al* Performance of rapid influenza H1N1 diagnostic tests: a meta‐analysis. Influenza Other Respir Viruses 2012; 6:80–86. 21883964 10.1111/j.1750-2659.2011.00284.x PMC3288365

[5] Hazelton B , Nedeljkovic G , Ratnamohan V *et al* Evaluation of the Sofia Influenza A + B Fluorescent Immunoassay for the rapid diagnosis of Influenza A and B. J Med Virol 2015; 87:35–38. 24838873 10.1002/jmv.23976

[6] Lewandrowski K , Tamerius J , Menegus M *et al* Detection of influenza A and B viruses with the Sofia analyzer. Am J Clin Pathol 2013; 139:684–689. 23596120 10.1309/AJCP7ZTLJCP3LLMA

[7] Lee C , Cho C , Woo M *et al* Evaluation of Sofia fluorescent immunoassay analyzer for influenza A/B virus. J Clin Virol 2012; 55:239–243. 22871494 10.1016/j.jcv.2012.07.008

[8] Simmerman J , Chittaganpitch M , Erdman D *et al* Field performance and new uses of rapid influenza testing in Thailand. Int J Infect Dis 2007; 11:166–171. 16798041 10.1016/j.ijid.2006.01.005

[9] Uyeki T , Prasad R , Vukotich C *et al* Low sensitivity of rapid diagnostic test for influenza. Clin Infect Dis 2009; 48:e89–e92. 19323628 10.1086/597828

[10] Weinberg A , Walker M . Evaluation of three immunoassay kits for rapid detection of influenza virus A and B. Clin Diagn Lab Immunol 2005; 12:367–370. 15753248 10.1128/CDLI.12.3.367-370.2005 PMC1065206

[11] Lucas P , Morgan O , Gibbons T *et al* Diagnosis of 2009 pandemic influenza A (pH1N1) and seasonal influenza using rapid influenza antigen tests, San Antonio, Texas, April–June 2009. Clin Infect Dis 2011; 52(suppl 1):S116–S122. 21342882 10.1093/cid/ciq027

[12] Faix D , Sherman S , Waterman S . Rapid‐test sensitivity for novel swine‐origin influenza A (H1N1) virus in humans. N Engl J Med 2009; 361:728–729. 19564634 10.1056/NEJMc0904264

[13] Balish A , Warnes C , Wu K *et al* Evaluation of rapid influenza diagnostic tests for detection of novel influenza A (H1N1) virus–United States, 2009. MMWR Morb Mortal Wkly Rep 2009; 58:826–829. 19661856

